# Ultrasound and Intrapleural Enzymatic Therapy for Complicated Pleural Effusion: A Case Series with a Literature Review

**DOI:** 10.3390/jcm13154346

**Published:** 2024-07-25

**Authors:** Riccardo Inchingolo, Simone Ielo, Roberto Barone, Matteo Bernard Whalen, Lorenzo Carriera, Andrea Smargiassi, Claudio Sorino, Filippo Lococo, David Feller-Kopman

**Affiliations:** 1 UOC Pneumologia, Dipartimento Neuroscienze, Organi di Senso e Torace, Fondazione Policlinico Universitario A. Gemelli IRCCS, 00168 Rome, Italy; riccardo.inchingolo@policlinicogemelli.it (R.I.); andrea.smargiassi@policlinicogemelli.it (A.S.); 2 Facoltà di Medicina e Chirurgia, Università Cattolica del Sacro Cuore, 00168 Rome, Italy; simone.ielo01@icatt.it (S.I.); roberto.barone01@icatt.it (R.B.); matteo.whalen01@icatt.it (M.B.W.); lorenzo.carriera01@icatt.it (L.C.); 3 Division of Pulmonology, Sant’Anna Hospital of Como, University of Insubria, 21100 Varese, Italy; 4 Thoracic Surgery, Fondazione Policlinico Universitario Agostino Gemelli IRCCS, Catholic University of the Sacred Heart, 00168 Rome, Italy; filippo.lococo@policlinicogemelli.it; 5 Section of Pulmonary and Critical Care Medicine, Dartmouth Hitchcock Medical Center, Lebanon, NH 03766, USA; david.j.feller-kopman@hitchcock.org

**Keywords:** lung ultrasound, intrapleural enzymatic therapy, urokinase, alteplase, t-PA, DNase, complex pleural effusion, empyema

## Abstract

Pleural effusion is the most common manifestation of pleural disease, and chest ultrasound is crucial for diagnostic workup and post-treatment monitoring. Ultrasound helps distinguish the various types of pleural effusion and enables the detection of typical manifestations of empyema, which presents as a complicated, septated effusion. This may benefit from drainage and the use of intrapleural enzyme therapy or may require more invasive approaches, such as medical or surgical thoracoscopy. The mechanism of action of intrapleural enzymatic therapy (IPET) is the activation of plasminogen to plasmin, which breaks down fibrin clots that form septa or the loculation of effusions and promotes their removal. In addition, IPET has anti-inflammatory properties and can modulate the immune response in the pleural space, resulting in reduced pleural inflammation and improved fluid reabsorption. In this article, we briefly review the literature on the efficacy of IPET and describe a case series in which most practical applications of IPET are demonstrated, i.e., as a curative treatment but also as an alternative, propaedeutic, or subsequent treatment to surgery.

## 1. Introduction

The evaluation of patients with pleural effusions and empyema can be challenging [1]. Chest ultrasound (US) is a valuable tool in the diagnosis and management of these pathologies, since it can guide in the choice of the best treatment, helping practitioners assess the sonographic characteristics of the fluid [1,2]. Chest tube placement may be insufficient if the pleural cavity has fibrinous adhesions, septations, and loculations, which are all easily identifiable by lung US. When the chest drainage placement procedure fails or the patient is not suitable for surgery, intrapleural enzymatic therapy (IPET) can be considered a safe and valuable treatment [1]. IPET is non-invasive and easily accessible in all clinical settings. If administered early, it breaks loculations and facilitates pleural drainage, avoiding the need for surgery [3,4].

Here, we describe five cases in which IPET has been successfully used for the treatment of complicated pleural effusions (CPEs) and empyemas either alone or in combination with thoracoscopy. In each case, we address the use of US as a guide for monitoring the pleural effusion, choosing a treatment, and evaluating its effectiveness. Then, we present an update on the role of IPET for the management of patients with a CPE.

## 2. Case Series

### 2.1. Chest US Examination Technique and Surgical Procedure

In the case series, we report the clinical data and US findings of patients treated at the Pulmonology Unit of Fondazione Policlinico Universitario A. Gemelli in Rome, Italy.

US examinations were performed using the MyLab 50 machine (Esaote, Genoa, Italy). Convex (with a frequency of two to five MHz) and linear (seven to thirteen MHz) probes were used, without presets. All ultrasonographic evaluations were performed by two pulmonologists with many years of experience in chest US (R.I. and A.S.).

Whenever possible, we performed the US examinations with the patients in the sitting position. Initially, the lung bases were examined; the probe was placed transversely in the intercostal space between the paravertebral line and the posterior axillary line. Gradually, placing the probe longitudinally, the examinations were performed up to the lung apices and laterally in the space between the axillary lines.

In addition to information regarding the type of PEff (complex septated) or empyema, the US investigation was aimed at a comprehensive evaluation of the explorable peripheral parenchyma and diaphragm [5]. Any information on interstitial syndrome, pleural thickening, visible nodularity on the patient’s diaphragm, diaphragmatic mobility deficit, or reduced muscle thickness was collected. In all cases, the scans were performed at the abdominal level to view the adjacent perihepatic, perisplenic, and perirenal areas to evaluate possible pleural involvement due to transdiaphragmatic fluid spread. Surgical indications were discussed in multidisciplinary meetings in which clinical baseline conditions and radiological and US features were analyzed to comprehensive assess the risk-to-benefit ratio of surgery. In our institution, almost all the oncological and non-oncological surgical procedures used the “uniportal VATS” approach (the less invasive technique available currently). A three to four cm long incision is usually made in the IV or V intercostal spaces along the middle axillary line [6]. Surgical maneuvers are performed using dedicated instruments that are guaranteed to reach all the areas of the pleural cavity. Finally, a chest tube (usually 24 Fr or 28 Fr) was inserted through the same incision. Table 1 summarizes the main demographic, clinical, and ultrasonographic characteristics, as well as the treatment and outcome, of the five cases.

### 2.2. Case Report 1

**Figure 1:**
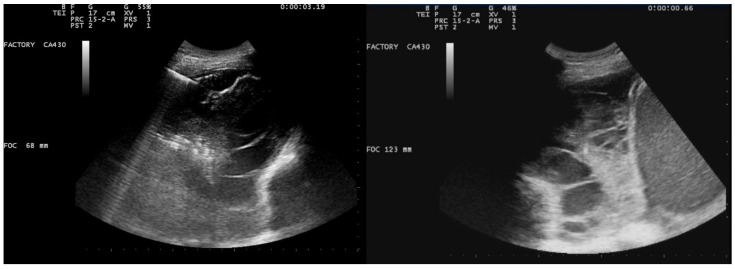
Chest US at admission. Multiloculated pleural effusion.

A 26-year-old man was admitted to our hospital for worsening dyspnea at rest and pain in the right hemithorax. The patient complained of a fever (39.5 °C) and productive cough for about 1 week, with mild hemoptysis in the days prior. His medical history included bilateral bronchiectasis and bronchial asthma, for which he received inhalation therapy with a long-acting antimuscarinic agent, long-acting bronchodilator, and inhaled corticosteroid. He had no family history of respiratory disease and had been tested for cystic fibrosis transmembrane regulator (CFTR) gene mutations, with negative results. The patient did not smoke cigarettes, drink alcohol, or use recreational drugs. An arterial blood gas (ABG) analysis showed acute hypoxemic normocapnic respiratory failure; therefore, oxygen therapy with a 28% FiO_2_ via a Venturi mask was administered. Chest radiography showed an area of opacification at the right base. Empiric antibiotic therapy with 12 mg/kg teicoplanine (Q8H) and 4.5 g piperacillin/tazobactam (Q6H) was then started on suspicion of community-acquired pneumonia. Blood tests upon his admission showed leukocytosis (WBC 18.84 × 10^9^ cells/L) with predominant neutrophils (14.5 × 10^9^ cells/L). Legionella and pneumococcal antigens in his urine were negative. Blood cultures after 72 h were negative. Chest ultrasonography revealed a multiloculated effusion (Figure 1).

**Figure 2:**
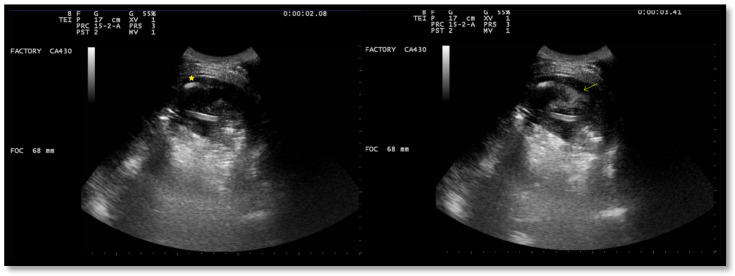
Chest US during pleural lavage with urokinase. Star: distal end of chest drain; Arrow: spread of fluid.

After the placement of a chest tube, pleural fluid samples were sent for microbiological and physicochemical analysis, which confirmed the diagnosis of pleural empyema. Two days after the placement of the chest tube, the fluid flow decreased. To break up the fibrin pockets present in the empyema and promote the complete evacuation of the fluid, irrigation of the pleural cavity with 200,000 UI of urokinase in 50 mL of saline solution was performed under US guidance (Figure 2).

**Figure 3:**
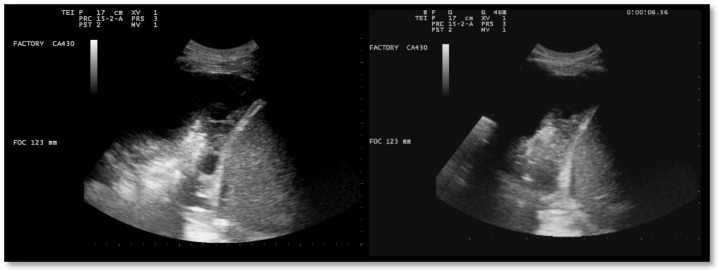
Chest US 48 h (**left**) and 72 h (**right**) after intrapleural instillation of urokinase.

Immediately following the instillation of the fibrinolytic agent, the chest drain was clamped for approximately 4 h. Then, irrigation with a few ml of saline solution was performed, and the chest tube was reopened. US observations 24 h after the intrapleural fibrinolysis showed a marked reduction in the extent of the effusion and localizations (Figure 3). After 72 h, the remaining fibrin sprouts had completely disappeared (Figure 3). This facilitated the drainage of the remaining exudate. At the same time, there was an improvement in his clinical condition with the disappearance of his fever and the restoration of normal oxygenation, which led to the discontinuation of oxygen therapy.

In this clinical case, intrapleural fibrinolysis was the only treatment that, in conjunction with antibiotic therapy, resulted in the resolution of the infectious scenario. Surgery was not necessary because, as the US showed, the effusion had probably just begun to form septa and loci, so treatment was given as early as possible. In addition, a prognostic key element was the observation of the parenchyma, which was atelectatic but was able to re-expand and retain some air bronchograms.

### 2.3. Case Report 2

A 76-year-old man was hospitalized for a fever and had had shortness of breath for 14 days. He was a former smoker of about 20 cigarettes/day and had quit smoking in 1985. He had no significant occupational exposures other than to smog (as a former traffic officer). He suffered from arterial hypertension, hypercholesterolemia, and chronic atrial fibrillation, for which he took warfarin. A physical examination revealed diffuse and bilateral chest crackles.

His chest X-ray showed the subtotal opacification of the left hemithorax, and his ABG test revealed significant hypoxemia with the following results: pO_2_ of 62 mmHg, pCO_2_ of 38.8 mmHg, pH of 7.52, and HCO_3_^−^ of 31.8 mmol/L. Therefore, he started oxygen therapy with a nasal cannula at 2 L/min.

**Figure 4:**
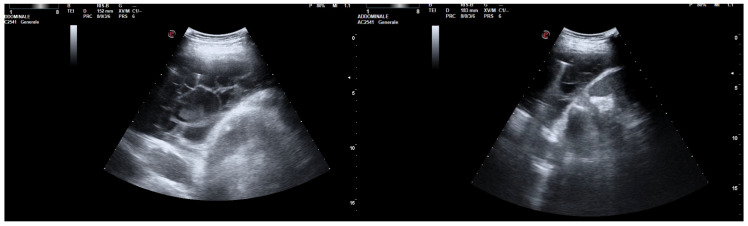
Complex and septated pleural effusion after VATS treated with IPFT.

After a chest CT scan, which showed a massive effusion, the patient underwent a thoracoscopic lavage of the left pleural cavity without any complications. At the end of the surgery, two left thoracic drains were left in place, through which repeated irrigation of the pleural cavity with urokinase was performed under serial US and radiological monitoring in the following days (Figure 4). At the same time, empiric antibiotic therapy with piperacillin/tazobactam and teicoplanin was initiated. During his hospitalization, the patient remained stably apyretic, with a progressive improvement of pulmonary gas exchange and the normalization of his inflammation biomarkers. Subsequently, the FiO_2_ was gradually titrated until the complete discontinuation of oxygen therapy.

In this scenario, fibrinolysis was performed after a uniportal VATS because of the persistence of fibrinous septa visualized using US and the slow outflow of fluid into the drainage bag. The irrigation of the pleural cavity was conducted with 200,000 UI of urokinase in 50 mL of saline solution. This was followed by the closure of the chest drain for 2–4 h, and then it was reopened. Irrigation was performed every 48 h for 3 days. On day 5, the absence of fibrinous septa and the small amount of fluid on the patient’s chest US allowed for the removal of the drain and his discharge from the hospital.

### 2.4. Case Report 3

**Figure 5:**
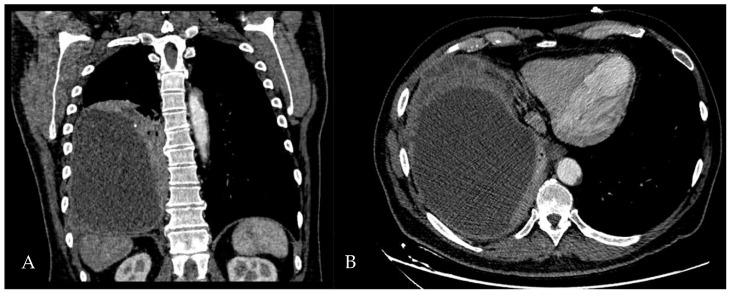
Chest CT scan of the mediastinal window in the coronal (**A**) and axial (**B**) plane, showing fluid collection in the medium–lower parts of the right hemithorax (maximum axial dimeters of 19 × 13 cm; craniocaudal extension of 16 cm) with thickened walls and contrast enhancement. There is also a complete atelectasis of middle and lower lobes and partial atelectasis of the upper lobe.

A 60-year-old Caucasian man was admitted to our hospital with a history of worsening pain at the base of the right hemithorax and feeling generally unwell for over one month. The patient’s past medical history included coronary heart disease, with a percutaneous coronary intervention in 2014. He smoked a half-pack of cigarettes per day for 40 years and had no history of respiratory disease. The CT chest scan showed a fluid collection in the medium–lower parts of the right hemithorax with thickened walls and contrast enhancement (Figure 5). This collection appeared in continuity with further smaller fluid collections with thickened walls, contrast enhancement, and confluence and caused the complete atelectasis of the middle and lower lobes and the partial atelectasis of the upper lobe, as well as middle and inferior lobar bronchus occlusion.

The patient’s blood tests upon his admission showed neutrophilia (11,950 cells/microL) and elevated levels of C-reactive protein (148.6 mg/L). A right chest drain tube was placed, and 1400 mL of purulent fluid were drained. Fluid samples were obtained for microbiological analysis, and empiric antibiotic therapy with 2 g vancomycin (Q12H) and 4.5 g piperacillin/tazobactam (Q8H) was started. An ABG analysis showed acute hypoxemic respiratory failure without hypercapnia; therefore, oxygen therapy with a 28% FiO_2_ via a Venturi mask was administered. After three days, the fluid samples taken from the chest drain tube resulted positive for *Bacteroides fragilis*, so the antibiotic therapy was maintained. At this point, one day after his admission, the patient was evaluated with a chest US, which showed a massive right PEff with echogenic material and impact on diaphragmatic kinetics. Therefore, a water seal was placed, the chest tube was opened, and 1000 mL of purulent liquid were drained. On the same day, the patient was evaluated by thoracic surgery to perform surgical drainage of the pleural cavity with a uniportal VATS.

The chest tube was left opened and drained under gravity. On day 3, the patient was re-evaluated with a bedside chest US, which showed a minimal basal viscous collection, the presence of organized air at the base of the pleural cavity because of non-expandable lung/hydropneumothorax, pleural thickening at the basal posterior site, and multiple fibrin ramifications branched with the diaphragmatic pleura, resulting in low inspiratory diaphragm motility. A thoracic surgical assessment was performed which confirmed the indication of surgery to clean the pleural cavity and restore diaphragmatic motility. The indication and the possible risks of the surgical intervention were discussed with the patient along with the thoracic surgeon. The patient, after full consideration of the risks, refused to undergo surgery.

On day V, following an evaluation with a chest US, the patient underwent an intra-pleural infusion of 200,000 IU urokinase in 50 mL of saline solution. The chest tube was then transiently closed and planned to be reopened after 2 h. On day 8, the chest tube drained little fluid, and another intra-pleural infusion of 200,000 IU urokinase in 50 mL of saline solution was performed. On day 10, a third intra-pleural infusion of urokinase was performed.

The patient’s clinical condition was stable, and a subjective feeling of improvement was referred to by the patient. On day 17, the chest tube was removed. During the following days, the patient was re-evaluated with a chest US, and no re-accumulation of material in the pleural cavity was shown. Minimal signs of the re-expansion of the previously consolidated parts were seen along with signs of diaphragmatic motility recovery. No fibrinous component inside the pleural cavity was shown. On day 19, the patient was discharged.

In this scenario, fibrinolysis was performed as an alternative to VATS because of the patient’s refusal to undergo the procedure. Fibrinolysis was successful in resolving the clinical picture and in restoring diaphragmatic motility even though this was several days after starting the medical treatment.

### 2.5. Case Report 4

**Figure 6:**
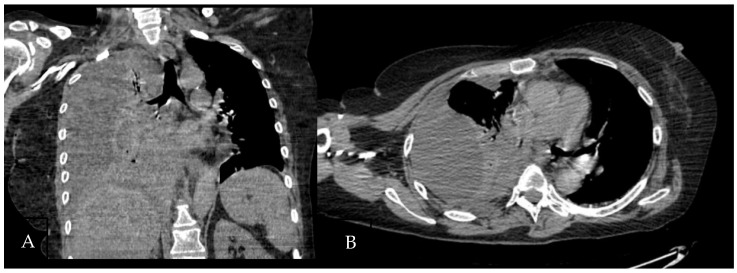
Chest CT scan of the mediastinal window in the coronal (**A**) and axial (**B**) plane, showing a large right pleural effusion, occupying almost the entire hemithorax with organized appearance. There is also a complete right lower lobe atelectasis and almost-complete right middle and upper lobe atelectasis with sparing of anterior sectors.

A 61-year-old female patient with obesity, discoid lupus, bipolar disorder, arterial hypertension, and hypothyroidism was admitted to our Respiratory Unit for respiratory failure and evidence of a large complex PEff on her chest CT, which also revealed complete atelectasis of the right lower lobe (RLL) and almost-complete atelectasis of the middle lobe (ML) and right upper lobe (RUL) (Figure 6). There was also a solid tissue surrounding the right main bronchus and its main branches, which was suspected to be neoplastic. It had a central hypodense area consistent with necrosis.

Peripheral enhancement of the pleural layers was also observed.

**Figure 7:**
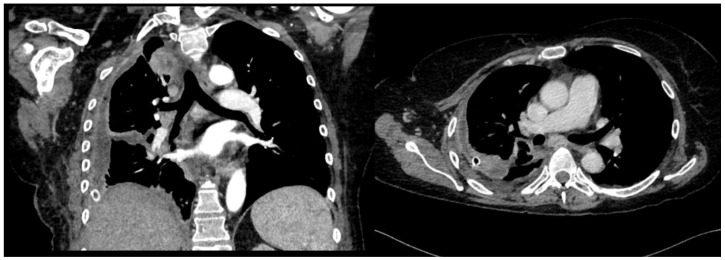
Lung re-expansion after IPFT and VATS.

A chest drain was placed, with the collection of purulent fluid. High-flow oxygen therapy was initiated, and the patient was treated with broad-spectrum antibiotics (vancomycin + piperacillin/tazobactam). The fluid was analyzed and was positive for *Streptococcus intermedius* and showed typical chemical and physical characteristics of exudates, so a diagnosis of a right pleural empyema was made. As a result, 3 days of treatment with intrapleural urokinase was initiated. The urokinase was administered in a daily single dose of 100,000 UI diluted in 50 mL of saline solution. At the end of the IPFT, a chest US was performed. It showed a decrease in effusion with a single loculation that extended over a width of three intercostal spaces. A chest CT was also performed, which confirmed the decrease in PEff and showed the re-expansion of RUL and ML and the partial re-expansion of RLL. The increased density formation described earlier was no longer evident. The patient was then referred for uniportal VATS to complete the toilette and obtain biopsies. Multiple pleural biopsies were performed. The results were consistent with chronic pleuritis, and no elements were noted that were suspicious in terms of malignancy. The biopsy showed fibrous tissue with hemosiderin-laden macrophages and scattered inflammatory cells. No neoplastic cells could be observed. Thus, a malignant lesion could be excluded. The chest tube was later removed, a chest CT was performed, and the patient was finally discharged (Figure 7).

This case describes the possible use of IPFT, before considering surgery, in a patient with a stage 2 fibrinopurulent empyema. Fibrinolysis was propaedeutic to VATS, which was performed to complete the decortication and obtain tissue samples. Indeed, patients receiving intrapleural fibrinolytic therapy require surgical referral in up to 10% of cases.

### 2.6. Case Report 5

**Figure 8:**
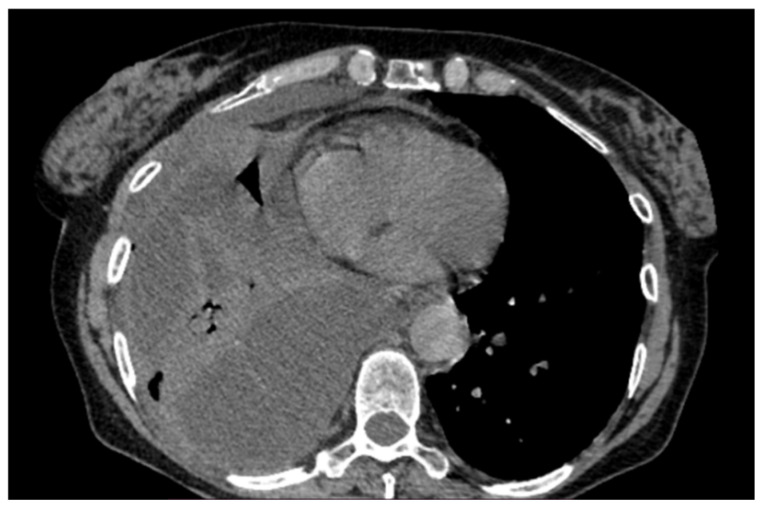
Large right pleural effusion on chest CT scan.

A 64-year-old Caucasian woman with iatrogenic hypothyroidism was admitted to the Respiratory Unit for worsening dyspnea, a fever, right chest pain, and coughing. The patient complained of having had a fever and dry cough for 1 week, treated with clarithromycin without improvement. Her pre-admission chest CT showed a large right PEff with lung atelectasis (Figure 8).

The patient was an active heavy smoker. Her past medical history included a thyroidectomy for a multinodular toxic goiter and hyperhomocysteinaemia treated with folic acid. She did not have a history of respiratory disease.

Her blood test upon her admission showed neutrophilia (14,550 cells/microL) and elevated levels of C-reactive protein (387.5 mg/L). A blood gas analysis revealed hypoxemia with hypocapnia (pH of 7.51; 60.8 mmHg paO_2_; 34.4 mmHg paCO_2_; 28 mmol/L HCO_3_^−^; 11 g/dL Hb). Her respiratory rate was 14 bpm, O_2_ saturation was 90%, and temperature was 36 °C. Oxygen therapy with a 28% FiO_2_ via a Venturi mask was started.

**Figure 9:**
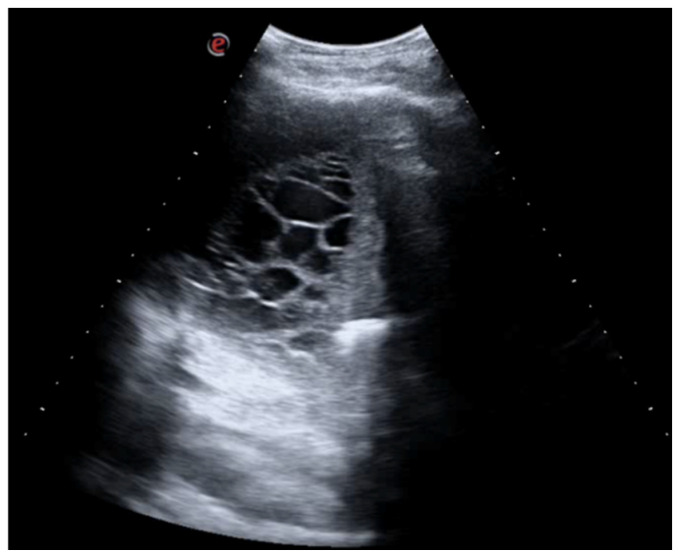
Chest ultrasound at admission showing multiloculated pleural effusion.

A chest US showed multiloculated PEff with septations of different thicknesses (Figure 9). A 12 Fr chest tube was placed, and 1000 mL of turbid fluid were drained. Samples were collected for microbiological and physicochemical analysis. Irrigation of the pleural cavity with 200,000 IU of urokinase in 50 mL of saline solution was performed under US guidance (Video 1). This was followed by the closure of the chest drain for 3 h, and then it was reopened. Further irrigation with 100,000 IU was performed 24 h later. Empiric antibiotic therapy with 500 mg azithromycin (q24h) for 6 days and 4.5 g piperacillin/tazobactam (q8h) for 2 weeks was administered. A chemical analysis of the pleural fluid revealed exudate. The microbiological study was negative.

**Figure 10:**
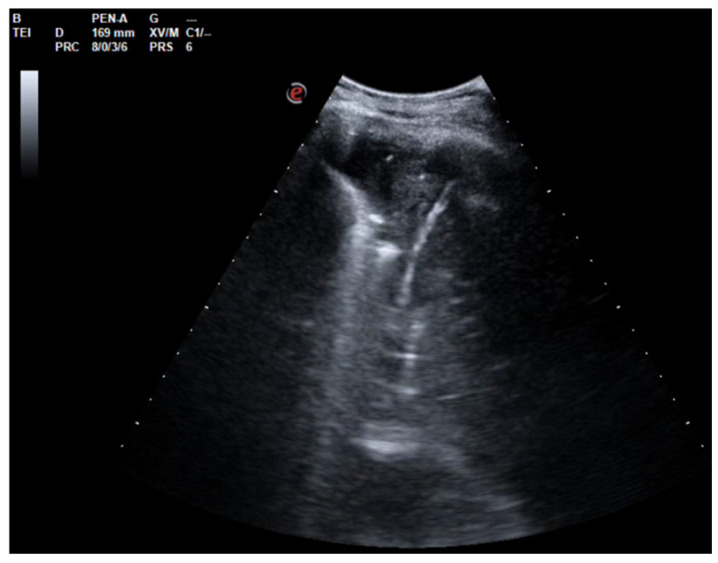
Chest US 48 h after urokinase. Minimum pleural effusion with residual fibrinous material and signs of lung re-expansion.

**Figure 11:**
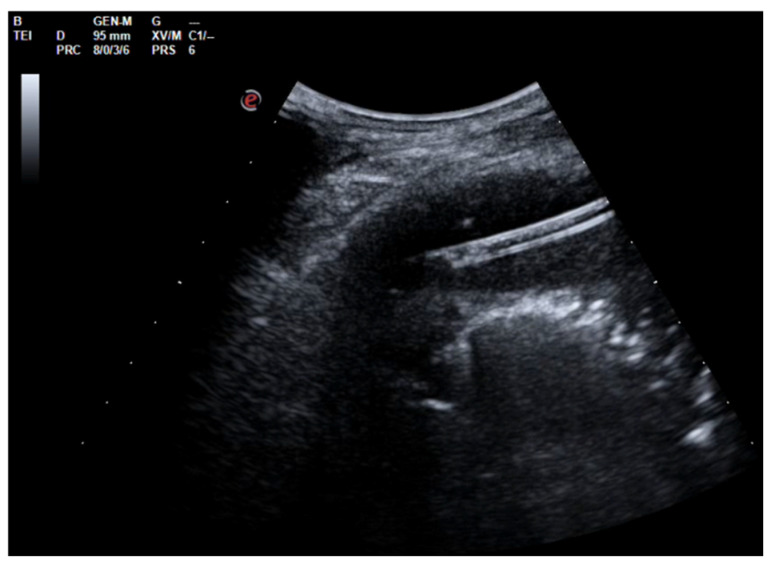
Chest US 48 h after urokinase. Chest tube in pleural cavity with residual pleural effusion.

Ultrasound observations 48 h after the administration of the first dose of urokinase showed the absence of fibrinous septa and a significant decrease in PEff (Figure 10 and Figure 11).

**Figure 12:**
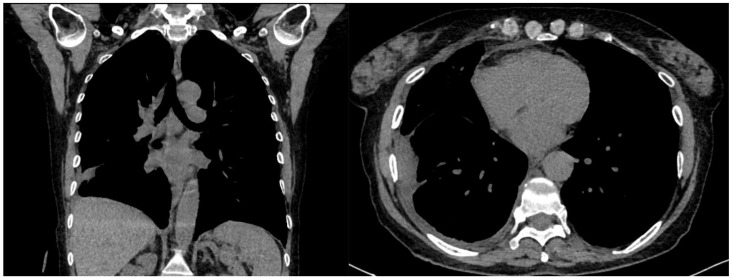
Pre-discharge chest CT.

During her hospitalization, the patient’s clinical conditions improved. A pre-discharge chest CT scan definitively ruled out malignancy (Figure 12). After 2 weeks, her chest tube was removed, and the patient was discharged.

In this case too, fibrinolytics and antibiotic therapy were the only treatments. The main clinical hypothesis was that she had a parapneumonic effusion that evolved into an empyema. Surgery was not necessary because of the progressive clinical and radiological improvement of the patient. The chest USs allowed for both a better characterization and a close follow-up of the PEff.

## 3. Materials and Methods

### Search Strategy

The literature research was conducted using the international database PubMed; the search strategy was not limited by time restrictions. Original articles, review articles, conference papers, and clinical trials were included in the evaluation. Only articles published in English were considered. Although the primary outcome of this study was to highlight the efficacy of IPET, other reported outcomes—such as articles on the pathogenesis of empyema and the role of chest USs—were equally evaluated. The process of data extraction and analysis was conducted independently by four authors (S.I., R.B., M.B.W., and L.C.) and was supervised by a fifth author (R.I.). The keywords used were as follows: “complex pleural effusion” OR “complicated pleural effusion” OR “empyema” AND “intrapleural fibrinolytic therapy” OR “intrapleural enzyme therapy” OR “intrapleural urokinase” OR “intrapleural alteplase” OR “intrapleural t-PA” OR “intrapleural DNase”. Finally, we selected articles in which the role of one or more fibrinolytic/enzymatic treatments were investigated and compared. Twenty-three articles were included in the review based on previously mentioned research keywords.

## 4. Pleural Effusion: The Role of Chest Ultrasound

A pleural effusion (PEff) represents one of the most common presentations of pleural pathology [1]. It can be primary, related to the direct affliction of the pleural layers, or secondary, related to systemic diseases.

The main distinction is between transudates and exudates. Light’s criteria have been commonly used for over 50 years for this purpose [7]. An LDH (or total protein) ratio of pleural fluid and serum > of 0.6 (or 0.5 for protein) is indicative of exudate.

The difference between these two types of PEffs is due to their pathogenetic mechanisms. Transudates originate in changes in hydrostatic pressure in capillaries or colloid osmotic pressure in plasma. The most frequent causes of PEffs include congestive heart failure, cirrhosis of the liver, and other causes of hypoalbuminemia.

Exudates usually arise because of increased permeability of alveolar–capillary membranes due to the action of inflammatory cytokines. Pneumonia, malignancy, pulmonary thromboembolism, and systemic inflammatory diseases are the most frequent causes of exudative PEffs [8].

Chest USs, in conjunction with patient medical histories, can predict with a good degree of confidence whether a PEff is transudative or exudative on the basis of its echogenicity and organization features [9,10,11,12]. Transudates are usually anechoic and non-septated (free flowing), whereas exudates can be anechoic or characterized by the presence of echogenic findings. The reverse, however, is not true. A hypoechoic effusion can be a transudate or an exudate, though hyperechoic effusions are typically exudates. The echogenicity within the pleural space occupied by the fluid can be homogeneous or heterogeneous. Additionally, an ultrasound can reveal fibrin, which presents as linear echogenic features attached at one edge and floating at the other. Fibrin strands can extend between the atelectatic lung and diaphragm or chest wall, thus creating a network within the pleural space, called septation or loculation (with thin and thick echogenic linear features, respectively). Such features indicate the presence of a CPE, also suggested by pleural fluid with a pH < 7.2, LDH content > 900 IU/L, or glucose content < 72 mg/dL.

CPEs typically require invasive procedures for their resolution, in addition to antibiotics [11]. The most important role of USs is distinguishing simple from complicated parapneumonic effusions [13].

The visible echogenicity in a PEff may be the expression of an active pathogenic process in the pleural tissue. In these cases, there are several possible explanations.

Inflammation of the visceral pleura adjacent to the lung parenchyma affected by pneumonia may result in a parapneumonic effusion, which occurs in 40 to 60 percent of patients with community-acquired pneumonia (CAP) [14]. The action of inflammatory cytokines (interleukine-8 and tumor necrosis factor–α) may lead to the loosening of the tight junctions between mesothelial cells in the visceral pleura, thereby promoting the passage of fluid from the pulmonary interstitium into the pleural space [15]. At this stage, referred to as the exudative stage [16], the pleural fluid is clear and contains neutrophils as can be seen by cell analysis, while bacteria are absent. At this stage, the selection of an appropriate antibiotic therapy based on risk factors for specific pathogens and local microbiologic resistance usually allows for the resolution of the pneumonia and the regression of the effusion [16,17]. The subsequent fibropurulent stage develops in cases of inadequate therapy or when inflammation proceeds abnormally. It is believed that about 10% of patients with CAP develop CPEs or pleural infections (i.e., empyemas) [14,16]. In this regard, some predictive risk factors have been suggested to identify the patients most susceptible to such complications early, including low albumin levels, hyponatremia, a high platelet count, elevated C-reactive protein, and a history of alcohol abuse or intravenous drug use [18].

Empyema is not always attributable to pneumonia and cases of pleural infection without adjacent lung consolidations are described in the literature [19,20], ascribed to hematogenous or transdiaphragmatic routes of spread. The fibrin stage is characterized by the progressive increase in bacterial translocation into the pleural space. The fluid is turbid or purulent; the metabolic activity leads to a decrease in pH and glucose. Increased local inflammation results in the maintenance of fluid production, and the molecules produced switch to a procoagulant phenotype. A central role is played by transforming growth factor-β (TGF-β), which is directly involved in fibrin production [21]. The cytokine microclimate that develops at this stage results in the disruption of the balance between the production and destruction of fibrin material due to the predominant activity of fibrinolytic molecules, such as the inhibitor of plasminogen activator (PAI-1) [22]. If the effusion is not adequately treated at this stage, it progresses to the organizing stage which is characterized by the proliferation of fibroblasts and the occurrence of loculations, in which fluid is trapped and drainage impracticable [16]. An organized effusion will affect the surrounding lung, which will become stiffer (“trapped lung”) and more difficult to re-expand even after treatment [16,23].

In this case, a visible US finding regarding the trapped lung is usually the absence of an air bronchogram as well as the thickening of the visceral pleura [24]. A comparative study by Cheng et al. analyzed US findings that may lead to a differential diagnosis of an abscess and an empyema, especially when the latter has a large air-fluid level [25]. Although passive atelectasis and septated effusions are specific to empyemas, the sensitivity was only about 40%. To increase the level of sensitivity, other features were examined, such as the shape of the lesion (spherical in the case of an abscess), the distance from the ribs and the diaphragmatic angle (an empyema is closer to these), and the Doppler signal in the parenchyma surrounding the lesion (which proved to be the most direct element in distinguishing an abscess from an empyema) [25].

Other signs may be recognizable in a CPE. Moreover, the effusion highlights the patient’s diaphragmatic profile and allows for the detection of otherwise invisible information, such as the presence of nodules or plaques (neoplasms and asbestosis) [24]. Pleural plaques are not indicative of malignant disease. However, they indicate exposure to asbestos and thus the possible risk of pleural mesothelioma. They are found bilaterally and along the entire pleural and pleuro-diaphragmatic surface, are larger at the base, are often calcified, and have hyper-echogenicity [26].

The hematocrit sign can be found in the hemothorax; it represents an area of effusion where the echogenic material (usually a corpuscular portion of the blood) collects in a inclined position due to gravity, and the anechoic fluid is directed upward [27].

In the context of an effusion, a fluctuating movement of hyperechogenic particles can be observed. This movement is called the “swirling pattern”, which is thought to be a predictor of malignant effusions [28].

## 5. Intrapleural Enzyme Therapy: State of the Art

The presence of loculations frequently makes chest drainage not completely effective in the treatment of an effusion. Streptokinase, urokinase, and alteplase (a recombinant tissue plasminogen activator: tPA) are fibrinolytic agents that have been used as an intrapleural treatment of CPE [29]. Recombinant Deoxyribonuclease (DNase) is an enzyme effective in reducing pleural fluid viscosity.

The three most important studies on intrapleural fibrinolysis are Multicenter Intrapleural Sepsis Trial-1 (MIST-1), Multicenter Intrapleural Sepsis Trial-2 (MIST-2), and Multicenter Intrapleural Sepsis Trial-3 (MIST-3) [Table 2]. The MIST-1 trial showed no benefit from therapy with streptokinase, the only one among the intrapleural fibrinolytics examined and compared to a placebo in 454 patients [30]. The MIST-2 trial documented that the combination of alteplase plus DNase improved pleural fluid drainage, reducing fluid viscosity, the need for surgical referral, and the length of patients’ hospital stays. However, the study also showed that alteplase alone or DNase alone was not effective in treating CPEs [31]. The MIST-3 trial is the first multicenter randomized controlled trial of early IPFT/IET versus early surgery for pleural infections. This study demonstrated an earlier resolution of pain and a shorter recovery time with IPFT/IET [32].

In 2019, Altmann et al. conducted a systematic review to determine which were the possible benefits and complications of intrapleural fibrinolysis in the treatment of CPEs and empyemas [33]. The review included ten randomized controlled trials (RCTs) comparing fibrinolytics with a placebo and two RCTs comparing different fibrinolytic agents (one trial compared treatment with streptokinase versus urokinase and the other one compared treatment with alteplase versus urokinase) [33]. The authors showed that fibrinolysis did not substantially reduce the risk of death compared to a placebo. Compared to placebo, fibrinolytics reduced the need for referral to thoracic surgery (open or thoracoscopic) and the overall rate of treatment failure (a combination of mortality and referral for surgery or additional fibrinolytic therapy) [33]. Both treatments with urokinase and alteplase plus DNase seemed to improve the clinical outcomes of patients with pleural infections [33]. They acknowledge the discordance between their conclusions and the randomized MIST-1 and MIST-2 data, which could be due to including smaller, non-randomized studies with an increased risk of bias.

Whether fibrinolytics should even be used is still being debated, along with the optimal dosing regimen of fibrinolytic agents, with different schedules reported in the literature. The 2021 consensus statement ultimately suggests that a trial of fibrinolytic plus DNase, injected concurrently, should be considered, even before surgery, in patients with suspected stage 2 empyemas and also makes recommendations on the appropriate dosing, dwelling time, and dose schedule [34]. The European Respiratory Society (ERS) and European Society of Thoracic Surgeons (ESTS) task force for the management of pleural infections in adults suggest considering the combination therapy of tPA with DNase as a “rescue” therapy [35]. It should be undertaken early (within 48 h after the placement of the chest tube and the start of antibiotic therapy) if there is clinical, biochemical, and radiological evidence of treatment failure, as it may be a potential surgery-sparing intervention.

Possible adverse events of both IPFT and IPET must also be considered. Chest pain is the most common side effect [36]. Bleeding is the most common serious adverse event caused by intrapleural fibrinolysis [33]. It might occur in the chest (intra or extra-pleural), or in the airways (haemoptysis). The systematic review by Altmann et al. did not point out a significantly increased incidence of bleeding compared with a placebo [33]. The risk of intrapleural bleeding with fibrinolytics appears to be increased in patients on systemic therapeutic anticoagulation medicine and in patients with coagulopathy [34]. Urokinase appears overall to be safer than other fibrinolytics: the intrapleural injection of urokinase resulted in a lower incidence of bleeding events compared to alteplase, but a possible dose-dependent relationship in the bleeding rate for alteplase should be considered [33,37]. A recent study by Bédat et al. suggested that urokinase therapy is as effective as t-PA/DNase therapy in patients with pleural infections, with similar clinical outcomes and fewer complications [38].

Chest USs can identify patients amenable to IPET. The detection of a loculation or septation of pleural fluid through US was one of the eligibility criteria of the study conducted by Davies et al. [39]. Chest radiography, contrast-enhanced CT scans, and pleural US scans were performed as per their protocol in the subjects enrolled in the trial [32]. Diacon et al. included patients in their study if pleural fluid aspiration revealed an empyema or a CPE (defined as a pH < 7.0 or pH < 7.2 and evidence of loculations on chest X-ray or US images) [33]. Misthos et al. [40] assessed all participants with CT scans, US scans, or both for the presence of loculations [34]. In the study carried out by Thommi et al. [41], a PEff was defined as complex and therefore was suitable for treatment if it was an exudate and the chest CT and US scans showed multiple loculations [35]. Explaining the reasons why their study had a one-year mortality rate lower than the one reported by the MIST-1 and MIST-2 RCTs, Aleman et al. [37] reported that USs were performed, and actually all the patients presented septations or loculations on their US or chest CT scans, even if only 82% of the effusions were assessed as loculated according to chest radiography [30]. In two studies, USs played a role in the evaluation of patients’ response to treatment and contributed to patients’ follow-up as well. In the study conducted by Bouros et al. [42], their inclusion criteria were having multiloculated CPEs or empyemas confirmed by CT images, US images, or both. The effectiveness of the intervention was assessed by chest radiography, US scans, and/or CT scans: success was defined as a 24 h pleural fluid drainage less than 50 mL after 3 days of treatment, without residual pleural fluid collection on chest imaging. As per their protocol, the instillation of the fibrinolytic could be repeated if the amount of collected pleural fluid was less than 50 mL within the last 24 h and a persistent effusion could be observed on US or CT images. All patients were followed for up to 30 months with clinical examinations, chest X-rays, and US or CT scans [36]. In a later study by Bouros et al. [43], the diagnosis of CPEs and empyemas was based according the current definitions and was confirmed with CT and/or US. As in the previous study, the response to treatment was assessed radiologically through chest X-rays, US scans, and/or CT scans. Analogously, regarding the follow-up, all patients were also followed-up with using US scans [37].

In two RCTs, MIST-1 [30] and MIST-2 [31], USs are not utilized in the protocol and were not used to assess the outcomes, as the radiographic evaluation of the size of the pleural fluid collection was provided only via chest X-rays. Tuncozgur et al. [44] radiologically evaluated improvements (reductions in effusion size) only through chest radiography and CT scans [38]. When considering IPFT/IPET, the recent consensus statement of 2021 by Chaddha et al. [34] recommends that, after a baseline chest CT scan, daily chest radiography, a US scan, or both should be used to assess the appropriateness and timing of intrapleural fibrinolytic therapy. With regard to the evaluation of patients’ response to treatment, this should be assessed clinically and radiologically (through both chest X-rays and US, because compared to the former, chest USs can identify smaller pleural fluid collection, and they detect septations better than CT) [34].

Intrapleural therapy is a recognized treatment option in the management of CPEs and empyemas. Despite this, surgery with pleural decortication and exudate lavage is still considered the first-line treatment at many centers, probably because of the methodological weaknesses of many of the studies reported above. As such, new clinical trials are needed, and a recent study by Wilshire et al. was conducted for this purpose: to develop an algorithm comparing IPET with tissue plasminogen activator plus DNase and surgical decortication [45]. The aim of the study was not to evaluate patient outcomes, which also showed a level of efficacy equal to that of the treatments, but to evaluate the feasibility and safety of conducting RCTs comparing the two methods in patients with complex pleural infections.

## 6. Discussion

The incidence of parapneumonic effusions and empyema has increased in recent decades [35,46]; furthermore, parapneumonic effusions and empyema are independent predictors of mortality [47].

IPET significantly improves fluid drainage as well as reduces the frequency of surgical interventions and the duration of patients’ hospital stays, and smaller studies suggest IPFT may be beneficial as well. IET has revolutionized the approach to patients with CPE/empyemas [1]. Although recent evidence suggests using dual therapy combining tPA and DNase as the preferred intrapleural treatment to reduce surgical hospitalizations, concerns have been raised regarding their potential increased risk of serious bleeding side effects, compared to the use of fibrinolytics alone [31,48].

Furthermore, due to the high cost and limited availability of dual therapy with tPA and DNase, fibrinolytic monotherapy with urokinase remains a widely used therapeutic option [49], particularly in resource-limited settings [50,51].

The main clinical applications of IPFT have been described in this clinical case series. In all cases, urokinase was used at a dosage of 200,000 IU diluted in 50 mL saline solution. The reasons for this were as follows: the drug’s better availability, better safety profile, and easier mode of administration than other fibrinolytics (once daily) [52].

Even though the European Association for Cardio-Thoracic Surgery (EACTS) guidelines of 2015 recommend the use of intrapleural fibrinolysis only in cases in which surgical intervention or ventilation are not tolerated by the patient [53], in daily clinical practice, the use of intrapleural drugs, although proposed more than 40 years ago, will become increasingly popular and not only as an alternative for patients who refuse more invasive treatments.

Indeed, the invasiveness of surgery has been remarkably decreased by the introduction of uniportal VATS, which has been largely demonstrated to be a less invasive approach compared to multiportal VATS and certainly less so than a thoracotomy [54].

This small case series aims to emphasize the versatility of using IPFT in the personalized management of patients with CPE/empyema.

Although the efficacy of IPFT has been questioned by several clinical trials such as MIST-2 and -3, future research is required to better define the optimal management of CPEs and empyemas in adults, to assess the correct timing of IPFT, and to optimize fibrinolytics dosing regimens. Likewise, the role of early VATS vs. IPET is currently being investigated in MIST-4.

In the present series, we have adopted a combined approach by performing both surgery and administering IPFT. Concerning the role of surgery, from a logical point of view, this should be always considered after the (partial) failure of IPFT, especially when there are no improvements in the patient’s general clinical conditions beyond the resolution of the radiological scenario. However, we found that when performing surgery after IPFT, the toilette of the pleural cavity appears to be easier to perform because usually the persistent purulent PEff is localized to few points. The same goes for the decortication that sometimes is necessary in stage III empyema and that could be also performed via uniportal VATS after IPFT.

In addition, we have reported that one might be able to combine surgery and IPFT in an alternative way by performing uniportal VATS upfront and completing the therapeutic plan with IPFT. Even though we cannot generalize this care algorithm, since this should be tailored and adapted in a case-by-case approach, we suggest considering this strategy.

In fact, uniportal VATS has a great advantage in that it is less invasive, and it allows us to place a large bore drain in the chest cavity under visual control. Because of this, we may assume that IPFT may be administered the day after surgery, and its effect could be maximized by the caliber and position of the chest drain.

Future studies are needed to consolidate the role of chest USs as a “guide” for intrapleural fibrinolysis in the management of CPEs and empyemas.

## Figures and Tables

**Table 1:** Main demographic, clinical, and ultrasonographic features, as well as treatment and outcome, of the case series.

Case Number	Age	Sex	Chest US Appearance	Diagnosis	Intrapleural Enzymatic Therapy	Dose	Surgical Referral
Case 1	26	Male	Multiloculated effusion	Empyema	Urokinase	200,000 UI	No
Case 2	76	Male	Multiloculated effusion	CPE	Urokinase	200,000 UI	VATS performed after IPET
Case 3	60	Male	Multiloculated effusion	Empyema	Urokinase	FD: 200,000 UI SD: 200,000 UI (72 h after the previous dose) TD: 200,000 UI (48 h after the previous dose)	No (refused by the patient)
Case 4	61	Female	Multiloculated effusion	Empyema	Urokinase	100,000 UI	VATS performed after IPET
Case 5	64	Female	Multiloculated effusion	Empyema	Urokinase	FD: 200,000 UI SD: 100,000 UI (24 h after the previous dose)	No

CPE: complicated parapneumonic effusion; FD: first dose; SD: second dose; TD: third dose; VATS: video-assisted thoracic surgery; IPET: intrapleural enzymatic therapy.

**Table 2:** Main randomized controlled trials on intrapleural enzymatic therapy.

Trial	Study Design and Intervention	Patients	Primary End Point	Secondary End Points	Main Results
MIST-1 2005 [30]	Double-blind trial Intrapleural streptokinase (250,000 IU twice daily for three days) vs. placebo	454	Died or needed surgical drainage at three months	Rates of death and surgery, radiographic outcome, hospital LOS	Intrapleural streptokinase does not improve mortality, rate of surgery, or hospital LOS
MIST-2 2011 [31]	Blinded, 2-by-2 factorial trial Double placebo, intrapleural t-PA and DNase, t-PA and placebo, or DNase and placebo	210	Change in pleural opacity on chest-X-ray on day 7	Referral for surgery, hospital LOS, adverse events	Intrapleural t-PA–DNase improved fluid drainage, reduced need for surgical referral, and shortened hospital LOS
MIST-3 2023 [32]	Prospective multicenter randomized controlled trial Standard care, early IET (t-PA–DNAse), or early VATS	97	Assessment of the feasibility of randomizing participants to the three arms of the study	Hospital LOS, frequency of readmission, requirement for repeat intervention, VAS scores of pain, QoL	Potential shortening of hospital LOS with early surgery; earlier resolution of pain and a shorter recovery time with IET

t-PA: tissue plasminogen activator; LOS: length of stay; VAS: visual analog scale; QoL: quality of life.

## Data Availability

Data are available on request from the corresponding author.
